# Androgen receptor modulation following combination exposure to brominated flame-retardants

**DOI:** 10.1038/s41598-018-23181-0

**Published:** 2018-03-19

**Authors:** Joubert Banjop Kharlyngdoh, Ajay Pradhan, Per-Erik Olsson

**Affiliations:** 1 Biology, Örebro Life Science Center, School of Science and Technology, Örebro University, SE-701 82 Örebro, Sweden; 2 Glomerular Disease Therapeutics Laboratory, Department of Internal Medicine, Rush University Medical Centre, IL-60612 Chicago, USA

## Abstract

Endocrine disrupting compounds can interfere with androgen receptor (AR) signaling and disrupt steroidogenesis leading to reproductive failure. The brominated flame-retardant (BFR) 1, 2-dibromo-4-(1, 2-dibromoethyl) cyclohexane (TBECH), is an agonist to human, chicken and zebrafish AR. Recently another group of alternative BFRs, allyl 2, 4, 6-tribromophenyl ether (ATE), and 2, 3-dibromopropyl 2, 4, 6-tribromophenyl ether (DPTE) along with its metabolite 2-bromoallyl 2, 4, 6-tribromophenyl ether (BATE) were identified as potent human AR antagonists. These alternative BFRs are present in the environment. The aim of the present study was to determine the effect of mixed exposures to the AR agonist and the AR antagonists at environmentally relevant concentrations. *In vitro* reporter luciferase assay showed that the AR antagonists, when present at concentration higher than TBECH, were able to inhibit TBECH-mediated AR activity. These AR antagonists also promoted AR nuclear translocation. *In vitro* gene expression analysis in the non-tumorigenic human prostate epithelial cell RWPE1 showed that TBECH induced AR target genes whereas DPTE repressed these genes. Further analysis of steroidogenic genes showed that TBECH up-regulated most of the genes while DPTE down-regulated the same genes. The results indicate that when TBECH and DPTE are present together they will antagonize each other, thereby reducing their individual effects.

## Introduction

Endocrine-disrupting chemicals (EDCs) are either manmade or natural substances present in the environment as well as in products such as food, furniture, flame-retardants and other consumer products either as impurities or as constituents. According to the U.S. Environmental Protection Agency^1^, an endocrine-disrupting compound may be defined as “an exogenous agent that interferes with the synthesis, secretion, transport, binding, action, or elimination of natural hormones in the body that are responsible for the maintenance of homeostasis, reproduction, development, and/or behavior”. Recently, according to the Endocrine Society, EDCs may be defined as “an exogenous chemical or mixture of chemicals that interferes with any aspect of hormone action”^2^. Even though EDCs affect a wide range of endocrine functions in different tissues and organs, they were originally thought to mediate their actions entirely through the nuclear hormone receptors such as estrogen receptors, androgen receptors (ARs), progesterone receptors, thyroid receptors and retinoid receptors^3^. However, recent studies have shown that apart from alterations of nuclear receptor functions, EDCs also exert their effects via non-steroid receptors, transcriptional activators and different enzymatic pathways that are involved in steroid biosynthesis/metabolism, as well as through epigenetic mechanisms^3,4^. Some of these environmental chemicals have been associated with poor semen quality, testicular dysgenesis syndrome as well as an increased risk of testicular and prostate cancer^5–7^.

The endogenous androgens play a vital role in the normal development and function of the male reproductive system, and mediate their actions via AR signaling^8,9^. Although EDCs affect androgen-dependent signaling pathways through numerous mechanisms, modulation of AR function is a major mechanism. The increasing number of cases with masculinization of wildlife populations^10,11^ as well as the increasing incidences of prostate cancer development and progression^6^ indicate an increased presence of androgenic substances in the environment.

Several studies across different taxa have shown that 1,2-dibromo-4-(1,2-dibromoethyl)cyclohexane (TBECH) negatively impact endocrine and reproductive systems^12–16^ and that TBECH activates the chicken, human and zebrafish ARs^12–14,16,17^. TBECH has furthermore been shown to up-regulate the prostate specific antigen (PSA), which is a biomarker for prostate cancer^13,14^.

Although the presence of the two structurally similar BFRs allyl 2,4,6-tribromophenyl ether (ATE) and 2,3-dibromopropyl 2,4,6-tribromophenyl ether (DPTE), and the metabolite 2-bromoallyl 2,4,6-tribromophenyl ether (BATE) has been detected in animals, so far few studies have investigated the biological effects of these compounds. Recently, using molecular modeling and *in vitro* assays we showed that ATE and DPTE along with its metabolite BATE were able to bind and inhibit the activity of the human AR thereby acting as AR-antagonists^18^.

Previously it has been shown that anti-androgens such as flutamide, linuron, procymidone, p,p’-DDE, vinclozolin and prochloraz result in disturbed penile developments and disrupt sexual function and spermatogenesis in mammals^5,19–21^. Other BFRs acting as AR antagonists, including polybrominated diphenyl ethers (PBDEs) and their metabolites and hexabromocyclododecane (HBCDD)^22^ have been shown to alter the expression of genes involved in steroidogenesis^23^. It has also been shown that exposure of rats, from postnatal days 23 to 53, to DE-71 result in a significant delay in puberty and a decrease in seminal vesicle and ventral prostate weight^24^. Another study on adult rats reported a decrease in the size of the lateral and ventral prostate, seminal vesicles and bulbourethral gland^25^. Recently, in a study on chicken hepatocyte it was reported that exposure to ATE resulted in down-regulation of vitellogenin 2 (*VTG2*) and aromatase (*CYP19A1*) expression, suggesting that it may reduce the estrogen levels in chicken^26^.

ATE, BATE and DPTE have been detected in indoor dust samples from Vancouver, Canada with ATE being detected in 81% of the samples at levels up to 52 ng/g while DPTE was identified in 43% of the samples at concentrations up to 1200 µg/Kg^27^. The highest concentration of DPTE recorded was 1940 µg/Kg in sewer systems^28^ that corresponds to 3.65 µM. In a study of emerging flame-retardants in indoor environments in Norway the presence of TBECH was detected with indoor air concentrations of 77.9 pg/m^3^ in households, and 46.6 pg/m^3^ in schools^29^. Both TBECH and DPTE has been detected in Arctic mammals. TBECH was found at concentrations of 1.1 to 9.3 µg/kg (0.003–0.022 µM) in Arctic Beluga blubber^30^ while DPTE has been detected in seal blubber at much higher concentrations, up to 470 µg/kg (0.9 µM)^31^. TBECH was also the most common BFR detected in indoor and outdoor air samples in a study performed in Stockholm, Sweden^32^. DPTE was detected in 5% of the samples at 210 ng/g in household dust from Belgium^33^. It has been reported that DPTE is present in 95% of biota samples from Greenland^34^. Interestingly, studies on environmental samples such as sediment, soil and sludge as well as house dust and fire station dust samples have reported the concurrent presence of TBECH along with ATE, BATE and DPTE^35–37^. Currently, there is no information on the combined effect of these two different groups of BFRs, one exhibiting AR agonistic properties and the other with AR antagonistic properties^12–14,16–18^. The detection of these BFRs in household dust samples suggests chronic mixed exposures of people to these compounds.

The present study was aimed at analyzing the effects of mixed exposure to these BFRs. We determined if the AR antagonists ATE, BATE and DPTE could inhibit the AR activating potency of TBECH and if this would result in altered AR nuclear translocation. Gene expression analysis was performed to determine the effects of mixed exposure to TBECH and DPTE on the transcript levels of genes involved in steroidogenesis in the non-tumorigenic human prostate epithelial (RWPE1) cell lines.

## Results

### ATE, BATE and DPTE reduce TBECH-mediated luciferase activity

**Figure 1:**
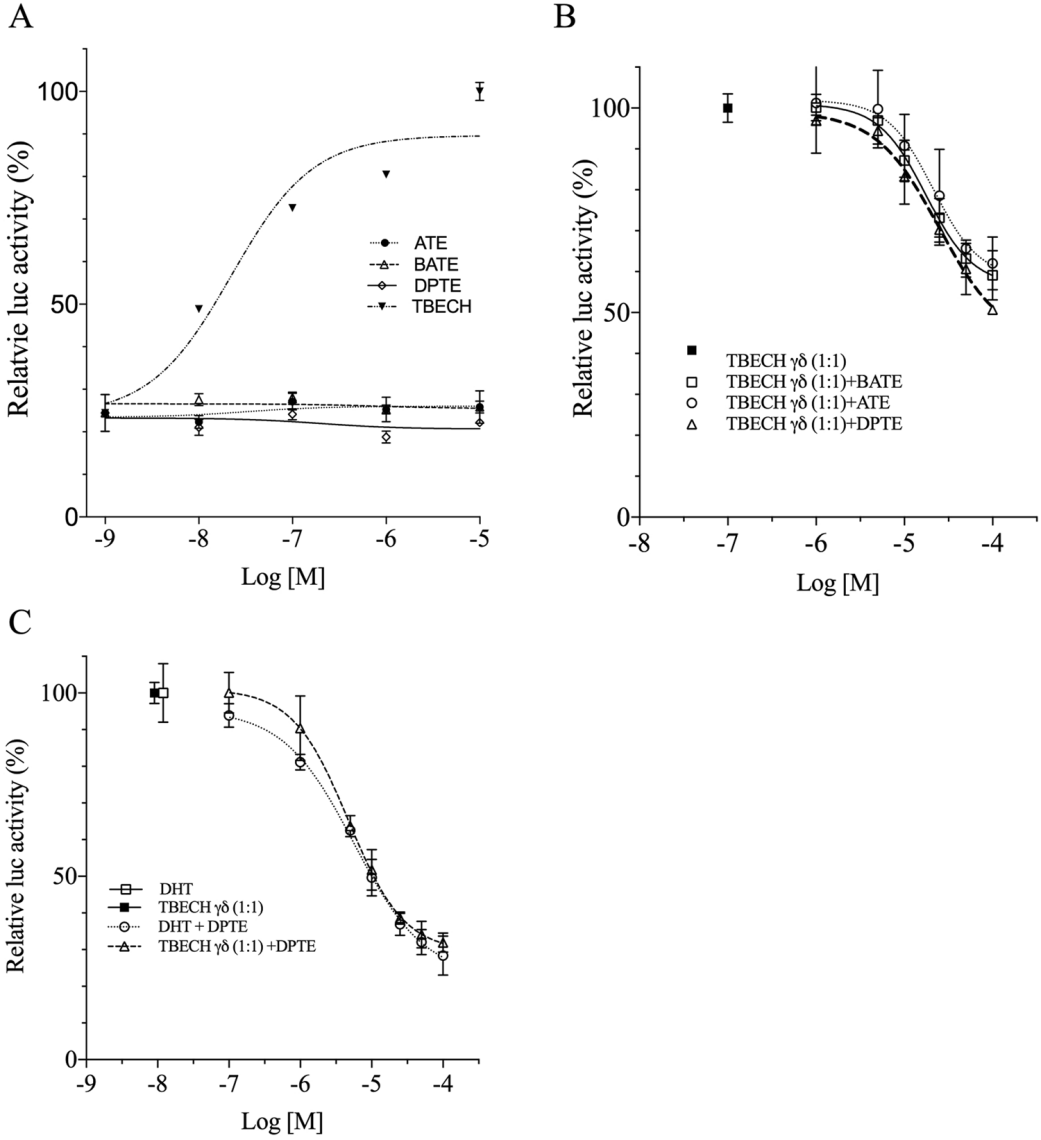
ATE, BATE and DPTE inhibit TBECH-γδ mediated luciferase activity. HeLa cells transfected with human androgen receptor expression plasmid were exposed to ATE, BATE, DPTE or TBECH in 0.1% DMSO (control) (**A**), 100 nM TBECH-γδ (1:1 mixture) alone or in combination with ATE, BATE and DPTE (**B**) and 10 nM each of DHT or TBECH-γδ (1:1 mixture) alone or in combination with DPTE for 24 hr (**C**). All values were normalized against the controls and the maximum response of TBECH was set to 100%. n = 4 per exposure group.

To determine whether ATE, and DPTE along with its metabolite BATE would reduce the AR activation by TBECH, transiently transfected HeLa cells were exposed to 100 nM TBECH-γδ (1:1) alone or in combination with different concentrations of ATE, BATE and DPTE. HeLa cells were chosen for these experiments as we have earlier observed that their slow metabolism (in contrast to HepG2 cells) of the endogenous steroids testosterone and DHT allows for co-exposure studies of TBECH, ATE, BATE and DPTE^13,14^. Exposure to ATE, BATE, and DPTE alone did not result in increased luciferase activity (Fig. 1A) confirming our earlier study showing that they are not AR agonists^18^. A significant decrease in TBECH-mediated luciferase activity was observed when cells were co-exposed to TBECH-γδ and ATE, BATE or DPTE. A 50% inhibition was observed with a 10,000-fold excess of the three BFRs (Fig. 1B). This demonstrates that all three BFRs are equivalent in potency at inhibiting TBECH-mediated luciferase activity.

To further determine the potency of DPTE at inhibiting DHT and TBECH-mediated AR activity, cells were exposed to 10 nM each of DHT and TBECH-γδ alone or in combination with different concentrations of DPTE. The results showed that DPTE was equally potent at inhibiting both DHT and TBECH-γδ mediated AR activity with 50% inhibition at 10.0 μM concentration (1000-fold excess) of DPTE (Fig. 1C).

### ATE, BATE, DPTE induces AR nuclear translocation

**Figure 2:**
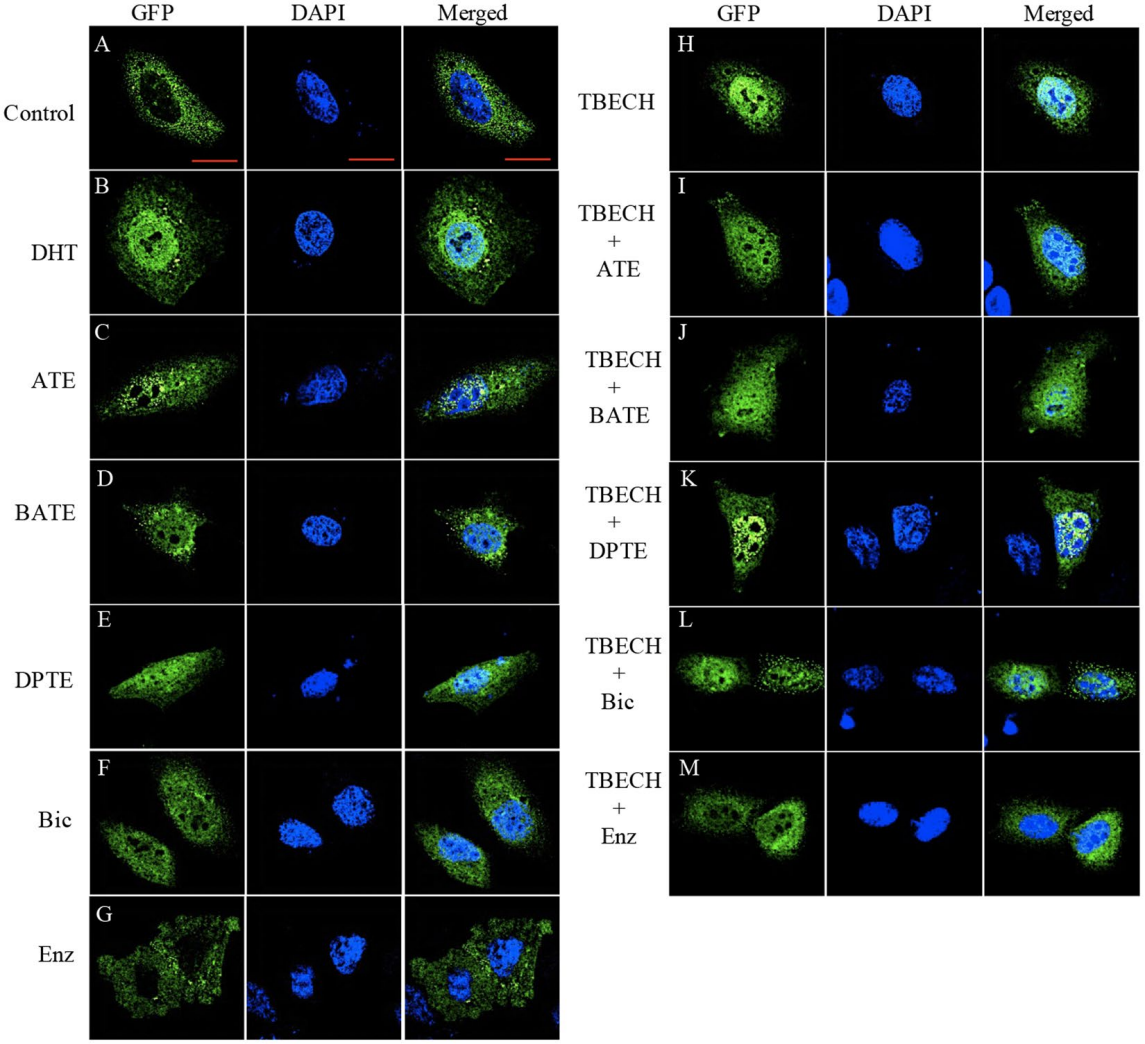
Subcellular localization of human AR following exposure to TBECH-γδ and different AR antagonists. HeLa cells transfected with plasmids pEGFP-hAR were exposed to 0.1% DMSO (control), 10 nM DHT, 100 nM of TBECH-γδ, and 10.0 µM each of ATE, BATE, DPTE, bicalutamide (Bic) and enzalutamide (Enz) alone for 2 hr. In addition, 10.0 µM each of ATE, BATE, DPTE, bicalutamide (Bic) and enzalutamide (Enz) was used in combination with 100 nM TBECH-γδ for another 3 hr. Cells were visualized by scanning confocal laser microscopy (60x magnification). Scale bar represents 20 µm.

ATE, BATE and DPTE inhibit DHT-mediated luciferase activity as well as TBECH-mediated luciferase activity in a dose-dependent manner. To further gain insight into the effect of ATE, BATE, and DPTE on the dynamics of AR regulation we studied the nuclear translocation of AR in HeLa cells transiently transfected with pEGFP-hAR. Initially, cells were exposed to 100 nM TBECH-γδ, and 10 μM each of the AR antagonists ATE, BATE, DPTE, bicalutamide and enzalutamide for 2 hr and sub-cellular localization was determined. Next, cells that were treated with the AR antagonists were co-exposed to 100 nM TBECH-γδ for an additional 3 hr and the sub-cellular localization was recorded. Most transfected cells exhibited exclusively cytoplasmic distribution of AR fused GFP protein in the absence of exposure (Fig. 2A). In cells treated with 10 nM DHT or 100 nM TBECH-γδ, the amount of AR fusion protein was almost exclusively located within the nucleus (Fig. 2B and H). Interestingly, exposure of cells to 10.0 μM each of the AR antagonists ATE, BATE, DPTE and bicalutamide alone resulted in distribution of AR to both the cytosol and the nucleus (Fig. 2C,D,E and F). However, following co-exposure of ATE, BATE, DPTE and bicalutamide (100-fold excess) with TBECH-γδ AR was exclusively localized within the nucleus (Fig. 2I,J,K and L). Exposure of cells to the second-generation AR antagonist enzalutamide alone resulted in predominantly cytoplasmic distribution of AR (Fig. 2G) that persisted in the presence of TBECH-γδ (Fig. 2M).

### TBECH-γδ and DPTE alter the transcription of androgen response genes in prostate epithelial cells

**Figure 3:**
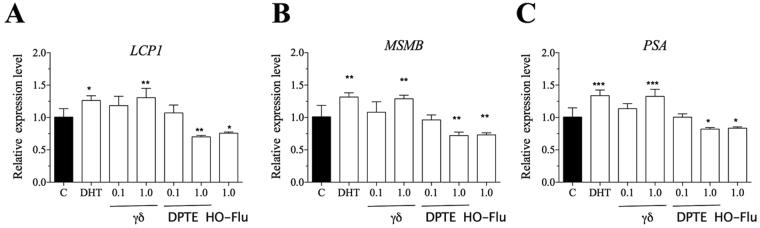
TBECH-γδ and DPTE alter the expression of androgen response genes. RWPE1 cells were exposed to 0.1% DMSO (control), 20 nM DHT, 1.0 µM of hydroxyflutamide (HO-Flu), and 0.1 and 1.0 µM each of TBECH-γδ (γδ) and DPTE for 24 hr. One-way ANOVA followed by Dunnett’s post-test for multiple group comparison was performed to determine statistical significance (*p ≤ 0.05; **p ≤ 0.01; ***p ≤ 0.001). Error bars represent Mean ± SD, n = 4.

**Figure 4:**
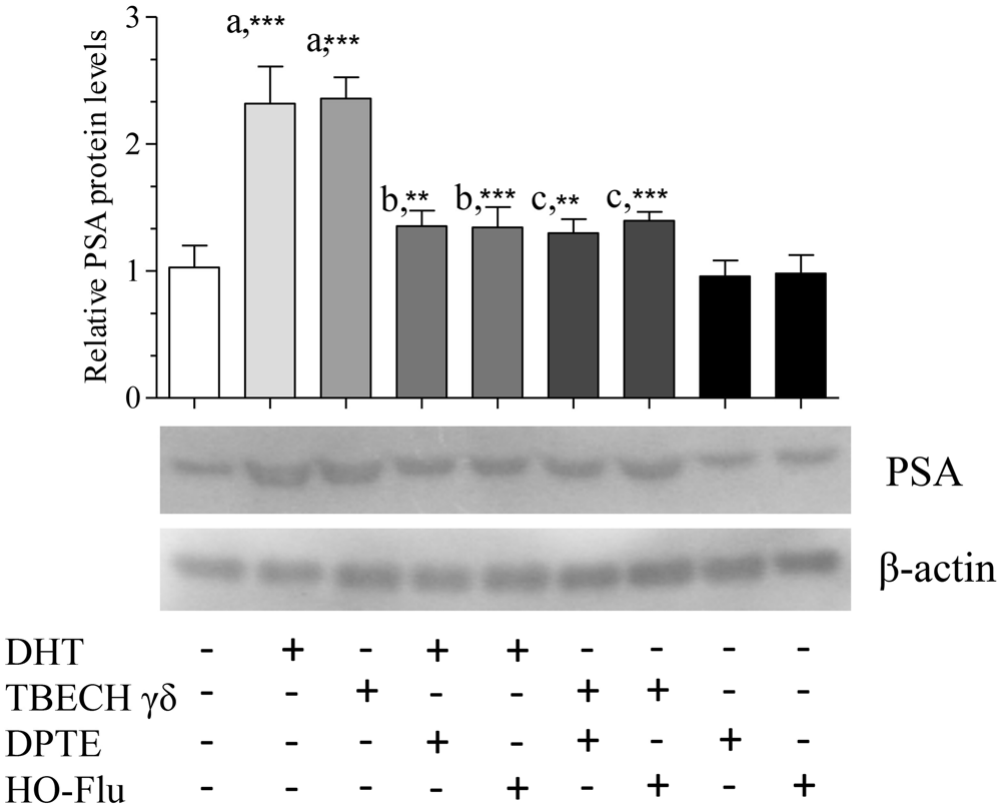
TBECH-γδ and DPTE alter the expression of prostate specific antigen protein. RWPE1 cells were exposed to 0.1% DMSO (control), 100 nM of DHT, TBECH-γδ and 20 µM of either hydroxyflutamide (HO-Flu) or DPTE alone for 6 days. For co-exposure experiments cells were exposed to 20 µM hydroxyflutamide and 20 µM DPTE together with 100 nM DHT or 100 nM TBECH-γδ for 6 days. PSA proteins were detected using PSA-specific antibody. β-actin was used as a loading control. The western blots results are represented by cropped gels and the complete western blots are shown in the supplementary material. One-way ANOVA followed by Dunnett’s post-test was used to determine statistical significance from (a) the control group, (b) DHT and (c) TBECH-γδ exposed cells (*p ≤ 0.05; **p ≤ 0.01; ***p ≤ 0.001). Mean ± SD of 3 independent experiments.

To analyze the effects of the BFRs on the expression of the AR target genes, RWPE1 cells were exposed to 0.1 and 1.0 μM of TBECH-γδ and DPTE, 20 nM DHT and 1.0 μM of hydroxyflutamide for 24 hr. The expression of the androgen response genes L-plastin (*LCP1*), microseminoprotein-β (*MSMB*) and prostate specific antigen (*PSA*) was up regulated by DHT and 1.0 μM of TBECH-γδ, while it was down regulated by 1.0 μM of DPTE and hydroxyflutamide (Fig. 3). These results were confirmed by analysis of PSA protein levels following exposure of RWPE1 cells to 100 nM DHT and TBECH that also resulted in significant increase of PSA protein levels (Fig. 4), a characteristic expected of androgenic compounds. Due to the low expression of PSA in the RWPE1 cells we used higher concentration of the compounds to follow the effects of co-exposure on PSA protein levels. Co-exposure of 100 nM DHT and TBECH with 20 µM of either DPTE or hydroxyflutamide resulted in significant decrease of PSA protein (Fig. 4).

### TBECH-γδ and DPTE alter the transcription of steroidogenesis genes in prostate epithelial cells

**Figure 5:**
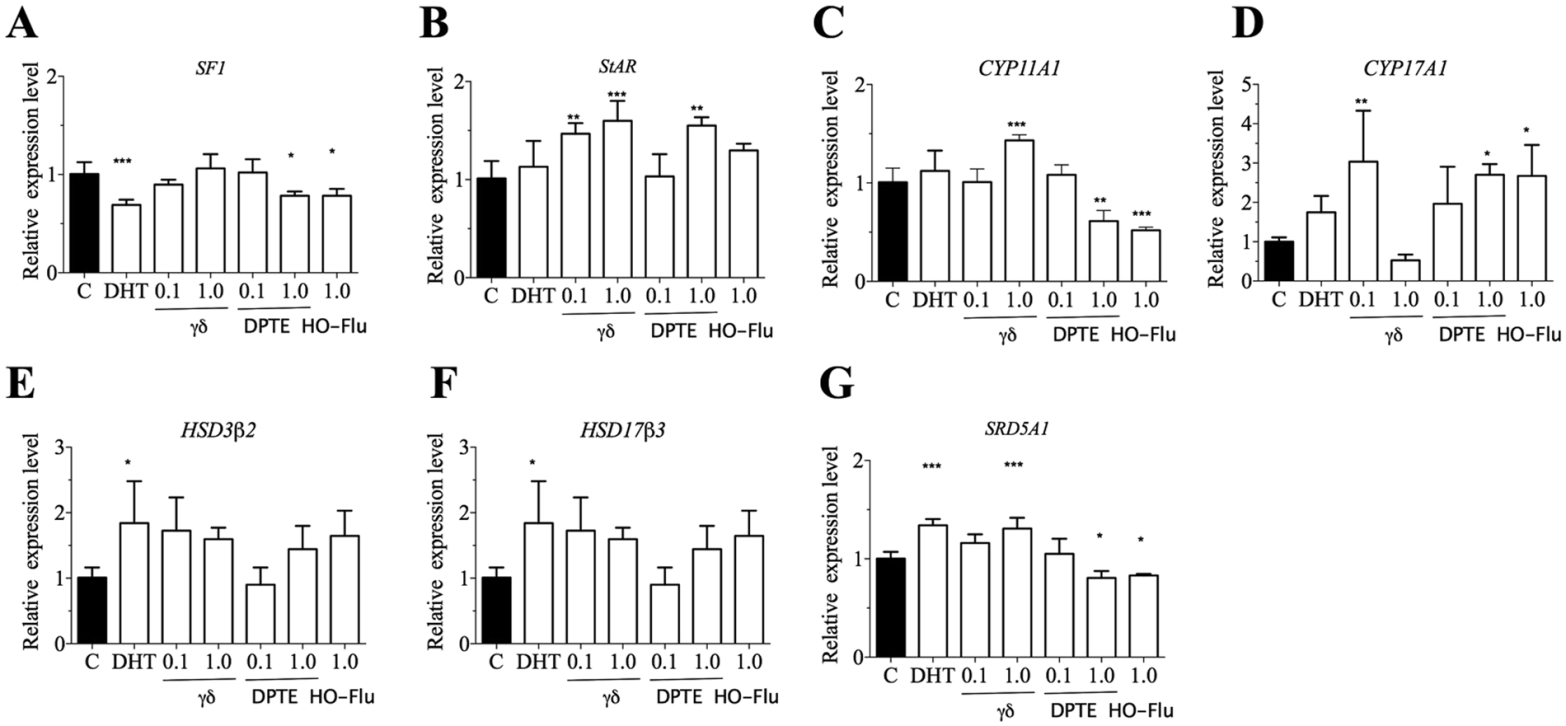
TBECH-γδ and DPTE alter expression of steroidogenesis genes. RWPE1 cells were exposed to 0.1% DMSO (control), 20 nM DHT, 1.0 µM of hydroxyflutamide (HO-Flu), 0.1 and 1.0 μM each of TBECH-γδ (γδ) and DPTE for 24 hr. One-way ANOVA followed by Dunnett’s post-test for multiple group comparison was performed to determine statistical significance (*p ≤ 0.05; **p ≤ 0.01; ***p ≤ 0.001). Error bars represent Mean ± SD, n = 4.

The effects of TBECH-γδ and DPTE on steroidogenic genes were analyzed. The expression of the steroidogenic factor 1 (*SF-1*) was down regulated following exposure to 20 nM DHT, 1.0 μM of DPTE and 1.0 μM hydroxyflutamide while it was not affected by TBECH-γδ exposure (Fig. 5A). The expression of the steroidogenic acute regulatory protein (*StAR*) was up regulated by 0.1 and 1.0 μM TBECH and 1.0 μM DPTE, but not altered following treatment with DHT and hydroxyflutamide (Fig. 5B). The expression of cytochrome P450 cholesterol side chain cleavage enzyme (*P450scc, CYP11A1*), which is involved in conversion of cholesterol to pregnenolone, was not altered by 20 nM DHT but significantly up-regulated by 1.0 μM of TBECH-γδ and down-regulated by 1.0 μM DPTE and hydroxyflutamide (Fig. 5C). Cytochrome P450 17α-hydroxylase/17, 20-lyase (*CYP17A1*) which is involved in the reactions converting pregnenolone to 17α-hydroxy-pregnenolone and then to dehydroepiandrosterone (DHEA), was not altered by DHT but up-regulated by 0.1 μM of TBECH-γδ, DPTE and hydroxyflutamide (Fig. 5D). In contrast, exposure to 1.0 μM TBECH-γδ resulted in a down regulation of *CYP17A1* indicating a dual effect on steroidogenesis. 3β-hydroxysteroid dehydrogenase/Δ(5)-Δ(4)-isomerase type 2 (*HSD3β2*), which is involved in multiple conversion reactions (pregnenolone to progesterone, 17α-hydroxy-pregnenolone to 17α-hydroxy-pregesterone, DHEA to androstenedione, androstenediol to testosterone) and 17β-hydroxysteroid dehydrogenase type 3 (*HSD17β3*) were up regulated by DHT but not by the BFRs or hydroxyflutamide (Fig. 5E,F). The expression of Steroid 5 Alpha- Reductase 1 (*SRD5A1*) was up regulated by DHT and 1.0 μM of TBECH but down regulated by DPTE and hydroxyflutamide (Fig. 5G).

## Discussion

Previously we have shown that the TBECH-γδ diastereomers are potent AR agonist for human^13,14,17^, chicken^12^ and zebrafish^16^ hence this mixture was selected for activation of AR-mediated luciferase activity in the present study. DPTE has been shown to be the most potent of the three BFRs at inhibiting DHT-induced PSA expression in LNCaP cells^18^. Thus, the presence of mixtures of these flame-retardants in the environment may result in a reduced response as they antagonize each other’s effects on AR mediated signaling in a dose dependent manner.

While being an AR antagonist, bicalutamide has been shown to exhibit AR agonistic properties at promoting AR nuclear translocation. However, it acts as an antagonist due to inefficient recruitment of co-activators resulting in an inactive transcription complex and inhibition of AR target genes^38^. Hydroxyflutamide also promote nuclear localization but retain its antagonistic properties by inhibiting AR dimerization and DNA binding^39^. The second-generation AR antagonist enzalutamide, which has been proposed to function as a pure AR antagonist inhibit AR nuclear translocation^40^. The present results suggest that ATE, BATE and DPTE, like bicalutamide or hydroxyflutamide, fail to block nuclear translocation of AR and therefore are weaker antagonists than enzalutamide.

To further analyze the effects of the studied BFRs we performed qRT-PCR analysis of genes regulated through AR. The *LCP1*, *MSMB* and *PSA* transcripts were analyzed. While DHT and TBECH-γδ up regulated all three genes, they were down regulated by the AR antagonists (ATE, BATE and DPTE). Human LCP1 is a leukocyte-specific actin-binding protein, was initially identified in transformed human fibroblasts, and later in normal hemopoietic cells and majority of human carcinoma cell lines^41^. *LCP1* expression is regulated by DHT in human prostatic carcinoma LNCaP cells^42^, and by testosterone in breast and prostate cancer cells^43^. MSMB is secreted by the epithelial cells of the prostate, is considered a prostate cancer marker^44^, and is up regulated by DHT and down regulated by DPTE in ductal breast cancer T47D cells^18^. The presence of *PSA* transcript in RWPE1 cells has been previously reported^45^ and its induction by DHT observed in this study was comparatively lower than that observed in LNCaP cells^18^. In a previous study on LNCaP cells we have also shown that DPTE inhibits the expression of *PSA* transcripts^18^. Over-expression of PSA protein is used as a biomarker in prostate cancer diagnosis and is also considered an important biomarker for androgenic activity^46^. This suggest that the AR agonist TBECH-γδ like the natural ligand DHT could play a crucial role in normal prostate growth, while on the other hand DPTE would counteract this effect.

Regulation of AR requires the synthesis of endogenous androgens from cholesterol. Analysis of steroidogenic gene regulation demonstrated that the BFRs altered the transcription of all genes involved in the conversion of cholesterol to DHT. DHT, DPTE and hydroxyflutamide down regulated *SF1*. *SF1* play a crucial role in regulating reproductive and endocrine functions including expression of steroidogenesis-related genes and is considered as a master regulator of steroid hormone production^47^. In contrast to DHT and hydroxyflutamide, TBECH-γδ and DPTE up regulated the *StAR* gene^48^. The transfer of cholesterol from the outer to the inner mitochondrial membrane is the first and rate-limiting step in steroidogenesis and is followed by conversion to pregnenolone by *CYP11A1*^49^. *HSD3β2* and *HSD17β3* that are involved in multiple conversion reactions were only affected by DHT.

*SRD5A*1 is involved in conversion of androstenedione to 5α-androstenedione and testosterone to DHT^50^. While *SRD5A1* was up regulated by DHT and TBECH-γδ, DPTE and hydroxyflutamide down regulated *SRD5A1*. It has been previously shown that *SRD5A1* is an androgen response gene and that its expression is up regulated by androgen treatment in prostate cancer cells^51^. In T47D cells, DHT induced the expression of *SRD5A1* while the AR antagonist DPTE repressed its expression^18^. The expression of cytochrome P450, family 19, subfamily A, polypeptide 1 (*CYP19A1*), involved in the conversion of testosterone to 17β-estradiol could not be analyzed due to low transcript levels (data not shown).

Previous studies have shown that exposure of animals to androgenic compounds result in masculinization and disruption of reproductive systems^10,52^ and this has also been suggested to increase the incidences of prostate cancer^6^. Studies with anti-androgens such as flutamide, linuron, procymidone, p,p’-DDE, vinclozolin and prochloraz have shown that exposure of mammals to these compounds result in induction of penile malformations as well as disruption of sexual function and spermatogenesis^5,19,20^. Prenatal exposure of female rats to testosterone has been shown to lead to permanently altered phenotype resulting in masculinization and presence of prostate tissue, usually not found in female rats^53^. In the present study, exposure of cells to the androgen DHT and AR agonist TBECH resulted in altered expression of genes involved in steroidogenesis. While TBECH-γδ up regulated *StAR*, *CYP11A1* and *SRD5A1*, it down regulated *CYP17A1*. In a recent study on porcine ovarian follicles, it was observed that testosterone up regulated the expression of *HSD3β2* while down-regulating *CYP17A1* expression^54^ indicating that there may be cell type specific and species-specific differences in the regulation of steroidogenic gene transcription.

In the present study, we show that exposure to TBECH-γδ and DPTE alter the expression of steroidogenic genes. TBECH-γδ induced the expression of genes involved in steroidogenesis while DPTE repressed these genes. This shows that environmental co-exposures of TBECH-γδ and DPTE may result in opposing effects of the two compounds masking the exposure to these compounds. There is clearly a need for further studies as these compounds may affect other systems and not necessarily have opposing functions in all systems.

## Materials and Methods

### Chemicals

**Figure 6:**
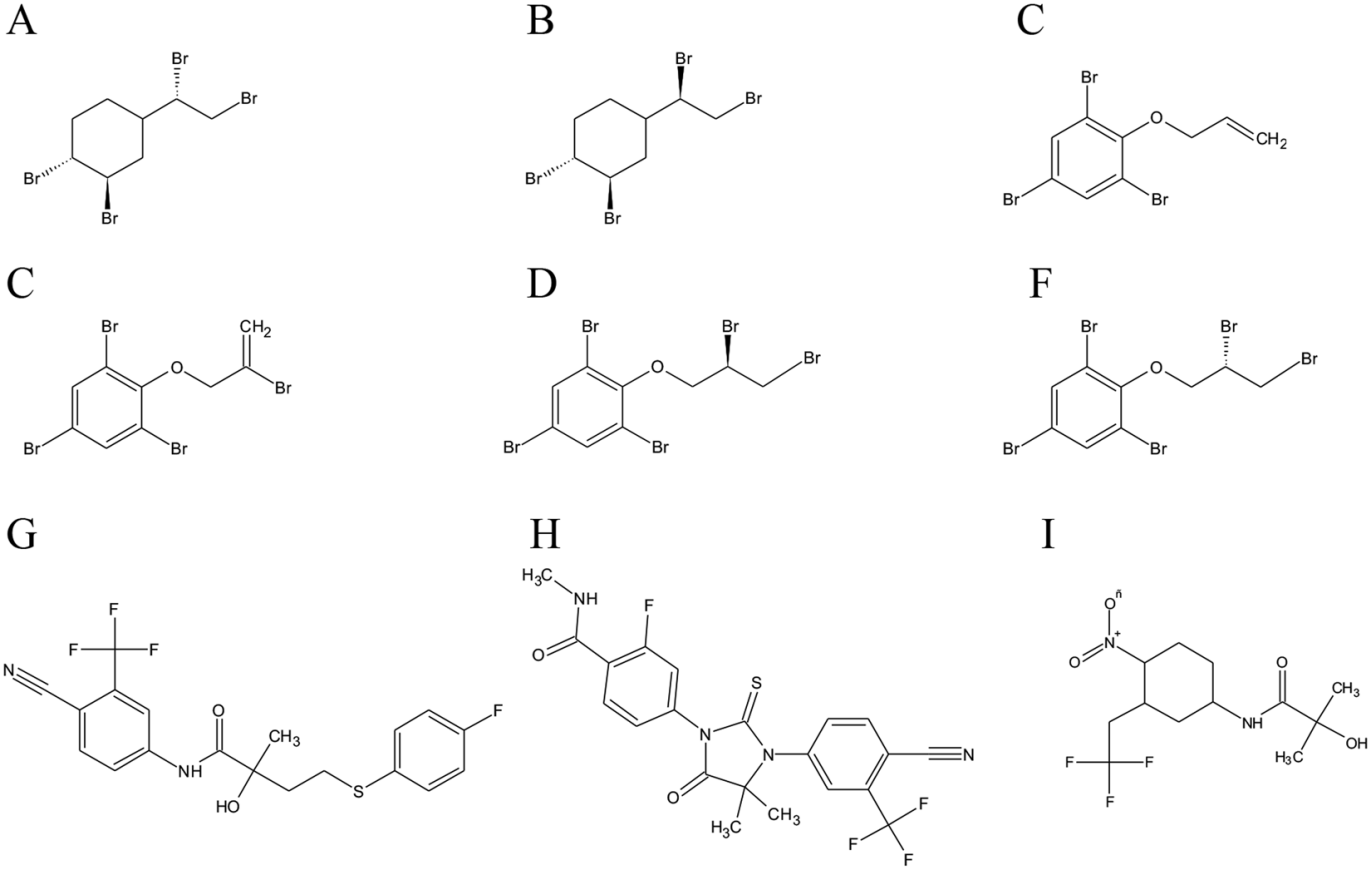
Molecular structures of the studied compounds. (**A**) TBECH-γ, (**B**) TBECH-δ, (**C**) ATE, (**D**) BATE, (**E**) DPTE-R, (**F**) DPTE-S, (**G**) Bicalutamide, (**H**) Enzalutamide, (**I**) Hydroxyflutamide. The full names and functions of the compounds are given in Table 1.

TBECH, ATE, BATE and DPTE were synthesized at 98% purity (Wellington Laboratories Inc., Canada) while bicalutamide, hydroxyflutamide (Sigma Aldrich, USA) and enzalutamide (Sellekchem, USA) were purchased. The studied compounds are described in Fig. 6 and Table 1. All the synthesized compounds, including bicalutamide and enzalutamide, were dissolved in dimethyl sulfoxide (DMSO). The amount of DMSO for exposure studies was maintained at a maximum of 0.1%.

**Table 1:** Description of compounds used in this study.

Compound	CAS No	Molecular weight	Uses
*rac*-(1R, 2R)-1,2-dibromo-(4R)-4-((1R)-1,2-dibromoethyl)cyclohexane (TBECH-γ)	3322-93-8	427.80	Expandable polystyrene beads
*rac*-(1R,2R)-1,2-dibromo-(4R)-4-((1 S)-1,2-dibromoethyl)cyclohexane (TBECH-δ)	3322-93-8	427.80	Expandable polystyrene beads
Allyl 2, 4, 6-tribromophenyl ether (ATE)	3278-89-5	370.87	Polyamide; polyester; polyethylene; polypropylene; polystyrene; polycarbonates
2-bromoallyl 2, 4, 6-tribromophenyl ether (BATE)	nd	449.76	nd
2, 3-dibromopropyl 2, 4, 6-tribromophenyl ether (DPTE)	35109-60-5	530.67	Polypropylene
Dihydrotestosterone (DHT)	521-18-6	290.44	Androgen/anabolic steroid
Bicalutamide	90357-06-5	430.37	Antiandrogen/prostate cancer drug
Enzalutamide	915087-33-1	464.44	Antiandrogen/prostate cancer drug
Hydroxyflutamide	52806-53-8	292.21	Antiandrogen/prostate cancer drug

### Cell lines maintenance

Human cervical carcinoma (HeLa) and the non-tumorigenic human prostate epithelial cell (RWPE1) cell lines were obtained from ATCC. HeLa cells were grown and maintained in DMEM medium (Hyclone, UK) containing 4 mM L-glutamine and supplemented with 10% FBS (Hyclone, UK). RWPE1 cells were cultured in a keratinocyte serum free medium (K-SFM, Invitrogen, USA), supplemented with bovine pituitary extract (5 mg/ml) (BPE, Invitrogen, USA) and human recombinant epidermal growth factor (5 ng/ml) (EGF, Invitrogen, USA), penicillin (100 U/ml) and streptomycin (100 μg/ml) (Invitrogen, USA). The cell lines were incubated in a stable environment of 95% humidity, 5% CO2, and 37 °C.

Prior to seeding, HeLa cells were grown for 48 hr in MEM phenol-free medium (Invitrogen, USA) complemented with 10% charcoal-stripped FBS (PAA Laboratories, Australia) as previously described^18^.

### Plasmids construction

The AR open reading frame (ORF) was PCR amplified using plasmid pCMVhAR^17^ as a template with primers containing Xho I and Bam HI sites (Table S1) and cloned into pZeoSV2 (Invitrogen, USA), a stable expression vector to generate the plasmid pZSVhAR. Using pEGFP-1 promoter reporter vector (Clontech, USA) as a template the EGFP ORF was PCR amplified with primers containing Nhe I and Bam HI sites (Table S1) then cloned into pZSVhAR and the resulting plasmid assigned as pEGFP-hAR. The plasmid constructs were confirmed by DNA sequencing (Eurofins, Germany).

### Transient transfection and reporter assays

Prior to transfection, HeLa cells were seeded (8 × 10^4^ cells/well) into 24 well plates (BD Falcon, USA) and grown until 90–95% confluence. Transient transfection was performed using Lipofectamine 2000 (Invitrogen, USA) according to the manufacturer’s instructions, using 270 ng of the human androgen receptor expression vector (pZSVhAR), 270 ng of the *slp*-ARE-Luc (*sex-limiting protein*–androgen response element–luciferase) reporter, and 60 ng *Renilla luciferase* (pRL; Promega, USA). At 24 h post-transfection, the media was replaced with phenol-free media complemented with charcoal-stripped FBS containing different concentrations of TBECH diastereomers TBECH-γδ, whereas for co-exposure experiments cells were exposed to ATE, BATE and DPTE (1.0–100 μM range) alone or in combination with 0.01 μM of DHT, or 0.01 and 0.1 μM of TBECH-γδ. After 24 hr of exposure, cells were then washed with PBS buffer (pH 7.4), lysed with passive lysis buffer and luciferase activity was measured using the Dual Luciferase Assay Kit (Promega, USA) in a TD 20/20 luminometer (Turner Designs, Sunnyvale, USA). The luciferase values thus obtained were normalized to the corresponding *Renilla* luciferase value.

### Gene expression analysis

RWPE1 cells were seeded (2 × 10^5^ cells/well) into 12-well plates (BD Falcon, USA). Cells were treated with 20 nM DHT, 0.1 and 1.0 µM of TBECH-γδ and DPTE, and 1.0 µM hydroxyflutamide for 24 hr. Total RNA isolation and qRT-PCR analysis were carried out as described previously^18^. The Ct values were normalized against elongation factor 1a (EF1A) and the data was presented as relative gene-expression as determined by the ΔΔCt method^55^. The primers used are listed in Table S1.

### Confocal laser microscopy

HeLa cells were seeded (6 × 10^4^ cells) in 24 well plates containing cover slips. Transient transfection was performed using Lipofectamine 2000 (Invitrogen, USA) as described above with 0.6 μg/well of pEGFP-hAR plasmid. At 24 hr post-transfection, the medium was replaced with fresh charcoal-stripped medium containing 10 nM DHT, 100 nM TBECH–γδ and 10 μM each of the AR antagonists ATE, BATE, DPTE, bicalutamide and enzalutamide. After 2 hr of exposure, cells that were pre-treated with AR antagonists were co-exposed with 100 nM of TBECH–γδ for an additional 3 hr. Following exposure, the coverslips were taken out and washed in PBS and mounted on microscope slides using Prolong Diamond Antifade Mountant containing DAPI (Invitrogen, USA). Cells were mounted on slides immediately after 2 hr of initial exposure and then at intervals of 60 min after addition of TBECH–γδ. Cells were viewed under FluoViewTM FV1000 Confocal Laser Scanning Biological Microscope (Olympus, Germany) and images were captured using 60x objective with oil immersion lens. The FV10-ASW viewer 2.0 software (Olympus, Germany) was used for processing the captured images.

### Immunoblotting

RWPE1 cells were seeded (4 × 10^5^ cells/well) into 6-well plates (BD Falcon, USA) and exposed to 100 nM of DHT and TBECH-γδ alone or in combination with 20.0 µM of either hydroxyflutamide or DPTE for 6 days. Cells were lysed in RIPA buffer and protein was extracted. Following quantification, the proteins were transferred onto PVDF membranes (Amersham Biosciences, UK) as described previously^18^. The membranes were rinsed for 30 min with Tris-buffered saline-Tween (0.1%) and incubated overnight at 4 °C with mouse anti-PSA antibody (Sigma, USA) at 1:500 dilutions. The secondary antibody, HRP-conjugated anti-mouse IgG (Amersham Biosciences, UK) was incubated for 1 hr at 1:5000 dilution at RT. Detection was performed using the enhanced chemiluminescent method (Amersham Biosciences, UK). The membrane was then stripped and probed for β-actin using mouse anti β-actin antibody (Sigma, USA). The bands were analyzed and quantified using ImageJ software (National Institute of Health, USA) and normalized with their respective β-actin level.

### Statistical analysis

Statistical analysis was performed using GraphPad Prism 5 software (GraphPad Software, USA) using one-way analysis of variance (ANOVA) followed by Dunnett’s post-test for multiple group comparison. Statistically significant differences were considered if the p values were <0.05 (*p < 0.05, **p < 0.01 and ***p < 0.001).

## Electronic supplementary material

Supplementary information
