# Supplementary material for: Androgen receptor modulation following combination exposure to brominated flame-retardants
Source: Sci Rep. 2018 Mar 19;8:4843. doi: 10.1038/s41598-018-23181-0 (PMC5859252; doi:10.1038/s41598-018-23181-0)

Androgen receptor modulation following combination exposure to brominated flame retardants

Joubert Banjop Kharlyngdoh, Ajay Pradhan and Per-Erik Olsson

Table S1. List of primers used in this study.

Gene	Gene type/ function involved		Primer Sequence (5’ to 3’)
CYP11A1	Steroidogenesis	*forward*	CTGCATCTTCAGTCGTCTGTCC
		reverse	GGTGACCACTGAGAACCCATTC
CYP17A1	Steroidogenesis	*forward*	TCCCCAAGGTGGTCTTTCTGAT
		reverse	GTGGACAGGGGCTGTGAGTTAC
eEF1a	Reference gene	*forward*	TCTGGTTGGAATGGTGACAA
		reverse	ACGAGTTGGTGGTAGGATGC
HSD3B2	Steroidogenesis	*forward*	CTGCTGCCTCTCTTTCACACAA
		reverse	AGAAAGTTCTGGTTGGGCCAGT
HSD17B3	Steroidogenesis	*forward*	CTGAAGCTCAACACCAAGGTCA
		reverse	CTGCTCCTCTGGTCCTCTTCAG
LCP1	Androgen response	*forward*	TTAACAGATACCCTGCCCTGCACA
		reverse	TTGAGATCTTGTCCACCGATGCCA
MSMB	Androgen response	*forward*	CAGCGTTGTGATCTTTGCCACCTT
		reverse	ACCCACAGGTGTAGAAACAAGGGT
PSA	Androgen response	*forward*	ACACAGGCCAGGTATTTCAGGTCA
		reverse	AGCACACAGCATGAACTTGGTCAC
SF1	Steroidogenesis	*forward*	GTCATCCCTTCCTCCTCTCC
		reverse	CAGAGGTCAGGTGGGGTAGA
SRD5A1	Androgen response	*forward*	GGGCATCGGTGCTTAATTTACCCG
	and steroidogenesis	*reverse*	CGTGAAGAAAGCAAAAGCCGC
StAR	Steroidogenesis	*forward*	GTCCCACCCTGCCTCTGAAG
		reverse	CATACTCTAAACACGAACCCCACC
AR		*forward*	CCCTCGAGGGATGGAAGTGCAGTTAGGGC
		reverse	CGGGATCCCGTCACTGGGTGTGGAAATAG
EGFP		*forward*	CTAGCTAGCTAGCGCCACCATGGTGAGCAAGG
		reverse	CGGGATCCCGGGACTTGTACAGCTCGTCCATGC

Supplementary figure 1

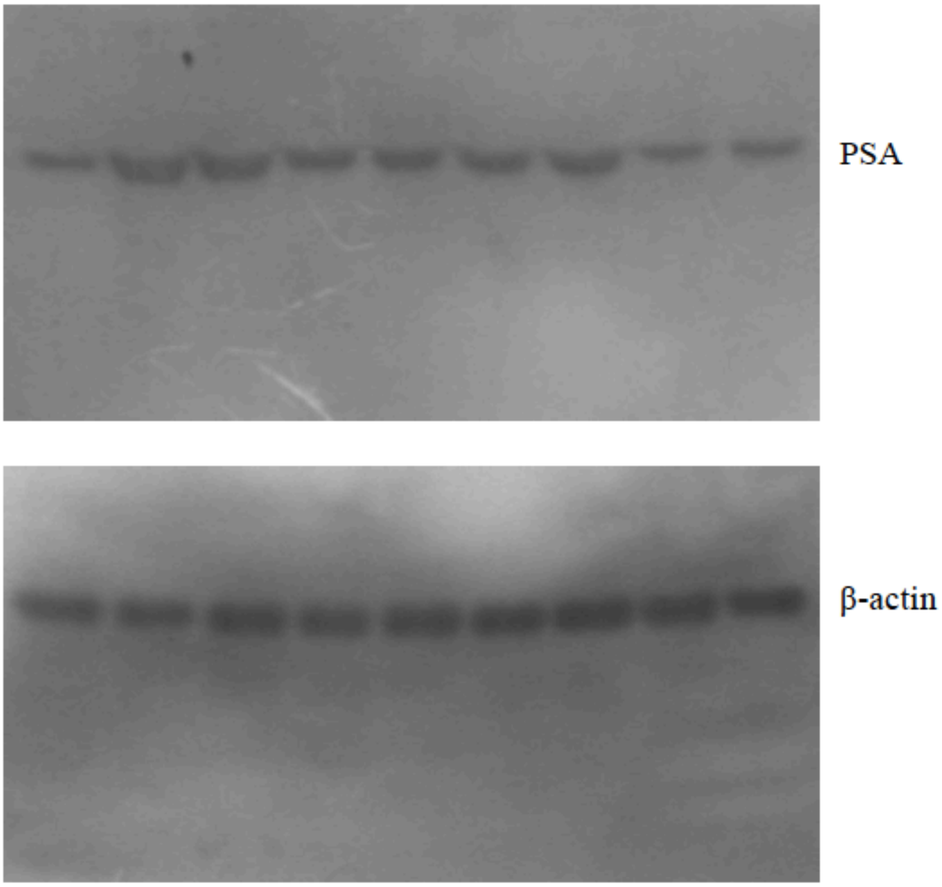

Supplement: Supplementary file 1 — Supplementary information [file 41598_2018_23181_MOESM1_ESM.pdf]
